# Oculocardiac Reflex During Intravitreal Injection

**Published:** 2020-03-22

**Authors:** Miguel Paciuc-Beja, Daniela Meizner-Grezemkovsky, Mario Paciuc, Idaira Sanchez-Santos, Anabeli Ruiz-Roman, Ashlee Fack, Andres Lisker-Cervantes, Gerardo Mendieta, Virgilio Morales-Canton, Hugo Quiroz-Mercado

**Affiliations:** 1 Ophthalmology Department, Centro Medico ABC, Av Carlos Fernandez Graef, Mexico City, Mexico.; 2 Retina Department. Asociacion para Evitar la Ceguera en Mexico, Vicente Garcia Torres, Mexico City, Mexico.; 3 Department of Statistics, Rice University, Houston TX, USA.

**Keywords:** Oculocardiac Reflex, Intravitreal Injection, Basal Heart Rate

## Abstract

Oculocardiac reflex (OCR) has been described to occur with mechanical manipulation of the eye, eyelids or orbit. There are no reports in the literature of OCR during intravitreal injection (IVI). This may be due to the fact that heart rate is not monitored during the procedure. We aimed to evaluate OCR during IVI. A total of 532 patients were enrolled in the study at Asociacion para Evitar la Ceguera en Mexico. Mexico City, Mexico. IVI was performed on one eye in every patient with diabetic retinopathy (DR), age related macular degeneration (AMD) or choroidal neovascularization (CNV) secondary to pathological myopia. Heart rate was monitored with a pulse oximeter before, during and after injection. OCR was defined as a 20% decrease or more of basal heart rate. The population enrolled included 270 females and 262 males with mean age of 63.8 years. A decrease in heart rate of 20% or more occurred in 18 patients during IVI (3.3%; 95% confidence interval 1.85% and 4.92%). OCR was asymptomatic in these patients. OCR occurred in 3.3% of our patients during IVI. Hence, OCR must be considered when performing IVI.

## INTRODUCTION

The oculocardiac reflex (OCR) is a sudden onset bradycardia that occurs sometimes during mechanical manipulation of the eye, eyelids or orbit. The afferent pathway comes from the ophthalmic branch of the trigeminal nerve. The efferent pathway is carried by the vagus nerve, which is accountable for the vagally mediated response of bradycardia [1-3]. Although sinus bradycardia is the most frequent effect of OCR, arrhythmia and asystole have been reported as well [4-7]. OCR occurs more frequently in children, especially during strabismus surgery [8-11] , it can also occur in adults [12, 13] and it has been described in a large variety of ophthalmic procedures [14-24].

The intensity of OCR decreases after repeated stimuli [25]. It is a fatigable reflex. Retrobulbar block [26], the use of anticholinergics [27-29] and deeper general anesthesia [10, 30, 31] can help reduce the occurrence of OCR. On the other hand, when fast-acting opioids [32, 33] or dexmedetomidine [34, 35] are used, they can increase OCR.

Intravitreal injection (IVI) of anti-vascular endothelial growth factor (VEGF) [36-40] and other drugs [41-44] is now the most frequent procedure performed in ophthalmology [45]. IVI of anti-VEGF is commonly used to treat a variety of retinal conditions, such as age related macular degeneration and diabetic retinopathy [39, 46-51]. It is estimated that 5.9 million IVI were performed in the United States in 2016 [45].

The purpose of this study was to evaluate OCR during IVI.

## MATERIALS and METHODS

**Figure 1:**
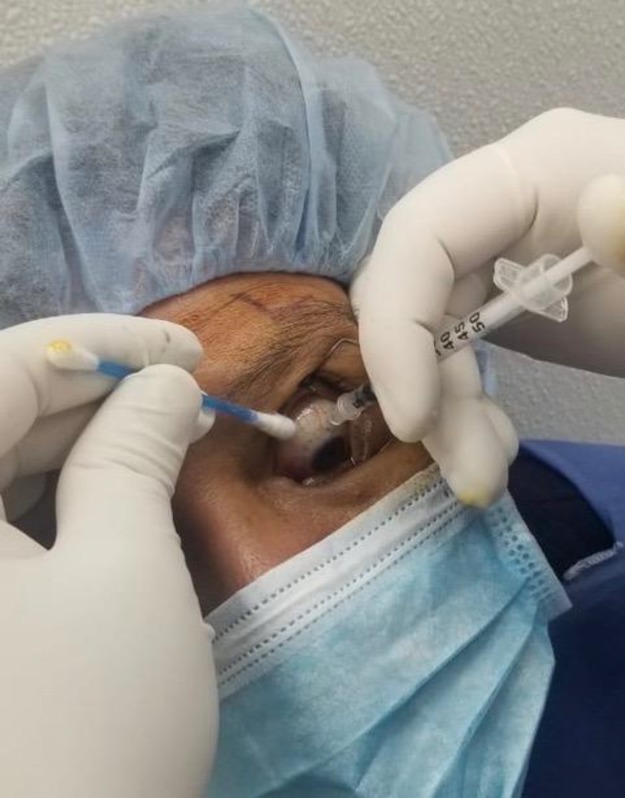
Superotemporal Intravitreal Injection in a Sterile Fashion in the Left Eye

During a 30-day period (June 2019), 532 patients scheduled for IVI were included for this study at the Asociacion para Evitar la Ceguera en Mexico (APEC) in Mexico City. The study complied with the Declaration of Helsinki. A written informed consent was obtained from all patients. This study received an ethical approval from our academic center. Only one eye of each patient was considered for the study. Patients taking medication for arrhythmia or having a pacemaker were excluded, all other patients were included. All injections were performed in the supero-temporal quadrant location. Injections were done following the 2018 consensus protocol recommendations [43]. The IVI was achieved in a sterile fashion (Figure 1). Heart rate was measured with a pulse oximeter (Zacurate, The USA) before and after placing the eyelid speculum, during the injection and after removing the speculum.

**Statistical analysis**

The percentage of patients with a decrease of 20% or more in basal heart rate was calculated using programming language R [52-54]. 

## Results

**Figure 2:**
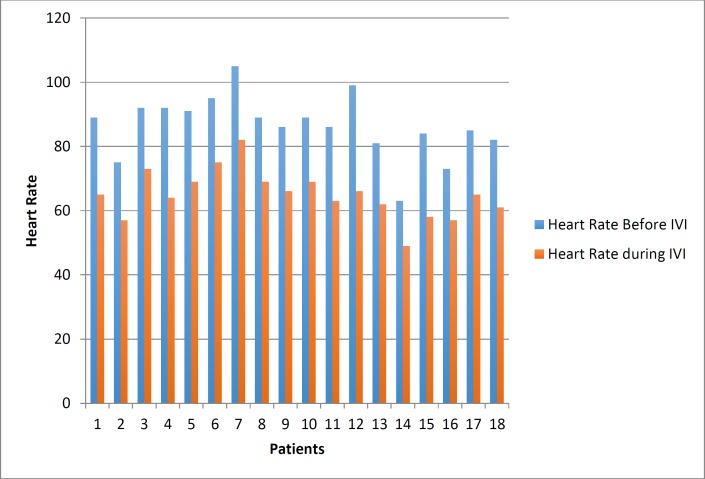
Basal Heart Rate (Heart Rate Before) and Heart Rate of Patients With Oculocardiac Reflex During Intravitreal Injection (IVI)

As shown in Table 1, of 532 eyes studied, 262 were males and 270 females, with a mean (range) age of 63.8 (29 – 89) years and 247 IVI in the right eye (OD) and 285 in the left eye (OS). Indications for IVI were respectively diabetic retinopathy (DR) in 352 eyes, age related macular degeneration (AMD) in 177 and choroidal neovascularization (CNV) secondary to pathological myopia in 3. In terms of the type of drug injected, 340 eyes received Ranibizumab (Lucentis; Genentech, The USA), 186 eyes Aflibercept (Wetlia; Bayer, Germany) and 3 eyes Ozurdex implant by IVI (Allergan, The USA; Dexamethasone intravitreal implant), respectively. Regarding OCR, of 532 patients enrolled, 18 presented a decrease in basal heart rate of 20% or more (Table 2 and Figure 2). As shown in Table 2, of 18 eyes, 12 were males and 6 females with a mean (range) age of 63.2 (41-84) years and 8 IVI in the OD and 10 IVI in the OS. Indications for IVI in these 18 patients were DR in 10 eyes, AMD in 6 eyes and CNV secondary to pathological myopia in 2 eyes. Ranibizumab was injected in 10 eyes and Aflibercept in 8 eyes. Statistical analysis showed a 3.3% incidence for OCR during IVI. A 95% confidence interval (CI) was found to be (1.85% and 4.92%). However, all patients with OCR were asymptomatic.

**Table 1.** Demographic Characteristics of the Study Subjects

Age; range (years)		29 – 89 (mean 63.8)
Gender; n	Male	262
	Female	270
Laterality; n	OD	247
	OS	285
Indication for IVI; n	DR	352
	AMD	177
	CNV	3
Injected Medication; n	Ranibizumab	340
	Aflibercept	186
	Ozurdex	3

Abbreviations: n: number of eyes; OD: right eye; OS: left eye; IVI: intravitreal injection; DR: Diabetic Retinopathy; AMD: age related macular degeneration; CNV: choroidal neovascularization secondary to secondary to pathological myopia; Ranibizumab: Lucentis (Genentech, The USA); Aflibercept: Wetlia (Bayer, Germany); Ozurdex: (Allergan, The USA) Dexamethasone intravitreal implant.

**Table 2:** A Summary of Characteristics of Patients With OCR During IVI

No.	Sex; F/M	Age; Y	Basal HR	IVI HR	Name of Medication	Side	Diagnosis
1	F	55	86	63	Aflibercept	OD	DR
2	F	84	91	69	Ranibizumab	OS	DR
3	F	63	89	65	Aflibercept	OD	DR
4	F	60	75	57	Aflibercept	OD	AMD
5	F	50	92	73	Aflibercept	OS	DR
6	F	59	85	65	Ranibizumab	OS	DR
7	M	76	92	64	Aflibercept	OS	AMD
8	M	41	95	75	Ranibizumab	OD	CNV
9	M	56	105	82	Ranibizumab	OD	DR
10	M	62	89	69	Ranibizumab	OD	DR
11	M	64	86	66	Aflibercept	OS	DR
12	M	54	89	68	Ranibizumab	OS	DR
13	M	60	99	66	Ranibizumab	OS	CNV
14	M	77	81	62	Aflibercept	OD	AMD
15	M	74	63	49	Ranibizumab	OS	AMD
16	M	65	84	58	Aflibercept	OS	DR
17	M	71	73	57	Ranibizumab	OD	AMD
18	M	67	82	61	Ranibizumab	OS	AMD

Abbreviations: F: female; M: male; Y: years; HR: heart rate; IVI: intravitreal injection; OD: right eye; OS: left eye; DR: Diabetic Retinopathy; AMD: age related macular degeneration; CNV: choroidal neovascularization secondary to pathological myopia; Ranibizumab: Lucentis (Genentech, USA); Aflibercept: Wetlia (Bayer, Germany).

## DISCUSSION

In this study, OCR occurred in 3.3% of patients during IVI. All patients who had OCR were asymptomatic. OCR occurred in patients of both genders and all were over 40 years. OCR occurred with both Aflibercept and Ranibizumab, both were the most frequent medications used in this study. 

OCR is more common in children, particularly during strabismus surgery, although other studies have shown that OCR can occur in adults [13]. Using electrocardiogram, in 1998 we published a series of adult patients with OCR during Laser-Assisted In Situ Keratomileusis (LASIK) [13]. In patients with OCR, decrease in heart rate appeared when the suction ring (pressure) was applied; all patients who underwent LASIK and presented a decrease in heart rate during the procedure were asymptomatic. 

For the purpose of this study, we measured how many seconds it took to perform the IVI. For the experienced ophthalmologist, IVI took 10 seconds or less, in contrast with rectus muscle traction during strabismus surgery (minutes) [55, 56].

OCR has been reported in 14% to 90% of patients during strabismus surgery [57]. Although OCR has been described in adult patients during different ophthalmological procedures [13, 20, 58], it is more common in children. Muscle traction during strabismus surgery and muscle entrapment in blowout fractures [59-62] of the orbit are the two most common circumstances associated with OCR. Individual cases of OCR during different procedures in adults under local anesthesia could be seen as anecdotal, but the ophthalmologist must be aware of its possible occurrence. 

We assume that in this study OCR was asymptomatic because of the short duration of the procedure [63-65]. The “2018 Update on Intravitreal Injections: Euretina Consensus Recommendations” addresses important issues related to pre-injection, peri-injection and post-injection management [45]. The authors stated that “the ophthalmologist should be aware of the potential cardiovascular and cerebrovascular risks of these agents”. Unusual systemic events after anti-VEGF IVIs have been reported, such as visual hallucinations, erectile dysfunction and acute decrease in kidney function [45] The role of anti-VEGF in these instances remains to be established.

After a comprehensive PubMed search, we could not find any report in the literature concerning OCR and IVI. This is because heart rate is not monitored during IVI and therefore not detected. A similar situation occurred during LASIK, OCR was not considered until heart rate was monitored during the procedure [13, 31]. This is the first report of OCR during IVI. Although our study included over 500 patients, it was single center, and compromised three types IVI and three medications. Therefore, further multicentric studies with more sample size, and wide range of injected medications and indications, must be done to confirm our findings. 

## CONCLUSION

In conclusion, OCR occurred in 3.3% of patients during IVI. Hence, OCR must be considered when performing IVIs.

## DISCLOSURE

Ethical issues have been completely observed by the authors. All named authors meet the International Committee of Medical Journal Editors (ICMJE) criteria for authorship of this manuscript, take responsibility for the integrity of the work as a whole, and have given final approval for the version to be published. No conflict of interest has been presented. Funding/Support: None. The datasets analyzed during this study are available from the corresponding author on reasonable request.
